# “Come and live with my feet and you’ll understand” – a qualitative study exploring the experiences of retail footwear in women with rheumatoid arthritis

**DOI:** 10.1186/s13047-019-0328-z

**Published:** 2019-03-14

**Authors:** Peta Ellen Tehan, Trish Morpeth, Anita Ellen Williams, Nicola Dalbeth, Keith Rome

**Affiliations:** 1 School of Clinical Sciences, Faculty of Health and Environmental Sciences, Auckland University of Technology, Auckland, New Zealand; 2 School of Medicine, Faculty of Medical and Health Sciences, University of Auckland, Auckland, New Zealand; 3 School of Health and Society, Univeristy of Salford, Salford, UK; 4 School of Medicine, Faculty of Medical and Health Sciences, University of Auckland, Auckland, New Zealand; 5 Department of Rheumatology, Auckland District Health Board, Auckland, New Zealand

**Keywords:** Footwear, Rheumatoid arthritis, Thematic analysis

## Abstract

**Background:**

Foot pain and deformity are common in people with rheumatoid arthritis (RA). Previous research has identified that women with RA seek retail footwear to alleviate their foot problems. The specific footwear features that women with RA require, and what would help them to find shoes that meet these requirements, are unknown. This study aimed to determine the factors that influence the choice of appropriate retail footwear by women with RA.

**Method:**

An overarching qualitative approach was taken, using reflexive thematic analysis of conversational style interviews. The interviews explored experiences and use of retail footwear in 20 women with RA. The interviews were digitally recorded transcribed verbatim and analysed using a reflexive thematic framework.

**Results:**

Women with RA sought retail footwear which had adequate cushioning, width, a flexible sole, lightweight, were made from breathable materials and were easy to put on and take off. However, this choice was driven by the need for comfort, cost and usability, with aesthetics being less of a priority. Despite having opinions on what criteria they felt that they needed, these women did not feel empowered to make good choices about purchasing retail footwear for symptomatic relief. Furthermore, they did not receive the necessary support from podiatrists and shoe shop staff.

**Conclusion:**

Women with RA have clear ideas about what features a retail shoe should have to achieve comfort. There is a constant compromise between achieving comfort and their feelings about their appearance and how they feel others perceive them. Women with RA describe negative experiences with shoe shop assistants and podiatrists leading to poor footwear choices. Both retail staff and podiatrists need increased understanding about the particular problems that women with RA experience.

## Introduction

Rheumatoid arthritis (RA) is characterised by inflammation of the synovium and destruction of joint architecture, leading to joint deformity and structural impairment [1]. High levels of foot pain and disability are reported [2] with approximately half of people with RA (53%) having foot involvement at diagnosis [3], and virtually all patients reporting foot problems within the first ten years of disease onset [4, 5]. The combination of structural change and foot pain results in difficulty for people with RA in sourcing suitable retail footwear that alleviates their symptoms [2]. Specialist therapeutic footwear has been shown to be of value, and central to the podiatric management plan in people with RA [6, 7], with previous research demonstrating that women with RA who utilise therapeutic footwear have improvements in pain and mobility. However, therapeutic footwear is often deemed unacceptable by women with RA due to aesthetics, price, or limited availability [8]. Furthermore, many women wearing therapeutic footwear alter their social behaviour and experience negative impact on body image and emotions [8].

Women with RA may seek retail footwear as an alternative to therapeutic footwear, but retail footwear may not be suitable, and can exacerbate their foot problems [9]. Naidoo et al. [10] demonstrated that women with RA face significant barriers in accessing appropriate retail footwear. Two previous studies [9, 10] have identified that for women with RA, purchasing shoes that meets the need for comfort and the desired aesthetics is challenging. This study aimed to determine the factors that influence the choice of appropriate retail footwear by women with RA using an overarching qualitative approach including reflexive thematic analysis. The results of this study will inform items to be used in the development of a patient reported assessment measure to assess the experience of footwear in women with RA.

## Methods

An overarching qualitative approach was taken, using reflexive thematic analysis of 20 conversational style interviews, to explore a range of different experiences in women with RA and their use of retail footwear. Participants were recruited from secondary care rheumatology clinics and the Auckland University of Technology Podiatry Rheumatology clinic in Auckland, New Zealand. Participants attended a single study visit at either Auckland DHB (Greenlane Hospital, Auckland) or Auckland University of Technology between March 2017 and August 2017. A targeted sampling strategy designed for maximal variation was used, to capture women of varying ages, ethnicity, RA disease duration, and employment status. This deliberate strategy was considered to be reflective of a generalizable population of women with RA in New Zealand. Ethical approval was granted by Health Disability Ethics Committee (HDEC 17/NTB/3) and written informed consent was gained from all participants.

Inclusion criteria for the study were: women aged twenty and above, RA according to the 2010 ACR/ EULAR classification criteria [11], able to speak English and provide written informed consent. Participants were excluded if they had a history of cognitive disorders or any lower limb amputation (minor or major). Interviews took place in a private clinical room. Interview questions were derived following a literature review of research relating to the experience of footwear in women with RA. Articles published between 1980 and 2017 and in the English language were included in the search strategy. The search was conducted using AMED, CINAHL, MEDLINE, Scopus, SportsDiscus and The Cochrane Library. Search terms used were footwear, foot problems and rheumatoid arthritis. Reference lists from retrieved publications were also examined to access additional articles. Five articles were deemed suitable [7, 12–15], with one study evaluating footwear use and the impacts of footwear in patients with arthritis using patient reported outcome measures, and four studies which developed a foot-specific patient reported outcome measure for use in arthritis. Based on a content analysis of items within these instruments, eleven questions were then formulated and an interview script prepared (Appendix 1). The use of an opening question “Tell me about your arthritis” was also included to put the participant at ease with the interviewer before focusing on the main questions. All interviews were digitally recorded and transcribed verbatim by a single researcher (TM) who de -identified all data to ensure participant confidentiality.

A reflexive thematic approach, a method described by Braun et al., was used to analyse the dataset whereby patterns of meaning (themes) are systematically identified and organised, providing insight across a dataset [16]. In accordance with a reflexive thematic approach, an additional researcher (PT) was firstly familiarised with the data through multiple readings of the entire dataset, becoming intimately familiar with the content. Through immersion in the data, patterns were recognised, and semantic and latent codes were generated by labelling important features of the data that may have been relevant to answering the research question. Codes were then collated and clustered to identify broader patterns of meaning and develop potential themes. Themes were then refined, and checked against the dataset, to determine that they tell a convincing story of the data, and if they answer the research questions. Themes were then constructed and defined by completing a detailed analysis, and determining the scope and focus of each theme, and finally themes were named. Data was managed using NVivo® software (QSR International©). Both researchers (TM and PT) were clinical podiatrists with experience of working with people with RA. An additional researcher (AW) provided oversight of the analytic process and assisted with the final coding framework and development of the themes. All authors reviewed and agreed on the final themes. Sample size was determined by the point at which it was determined that data saturation was achieved, which was the point at which the researchers deemed that collecting more data had no further interpretive value, and further coding was no longer feasible.

## Results

Twenty participants aged between 27 and 75 years old were interviewed. The range of RA disease duration was between 3 months to 45 years. Individual participant characteristics are in Table 1. The following themes were elicited: “Comfort’s number one”,“I don’t want to wear Nana shoes, and so many comfy shoes are Nana shoes” and “Come live with my feet and you’ll understand”.

**Table 1:** Individual Participant Characteristics

ID#	Age (years)	Disease Duration (years)	Ethnicity	Employment status	Recruitment Site
1	44	6	NZ European	Unable to work	Rheumatology clinic
2	56	6	NZ European	Employed	Rheumatology clinic
3	71	22	NZ European	Employed	Podiatry clinic
4	70	45	NZ European	Employed	Rheumatology clinic
5	64	29	NZ European	Retired	Rheumatology clinic
6	57	25	Asian	Retired	Rheumatology clinic
7	75	10	NZ European	Retired	Podiatry clinic
8	65	34	NZ European	Retired	Podiatry clinic
9	52	10	Pacific Island	Unable to work	Rheumatology clinic
10	27	2	Māori	Employed	Rheumatology clinic
11	72	3	Pacific Island	Retired	Rheumatology clinic
12	52	17	Pacific Island	Employed	Rheumatology clinic
13	40	6 months	Pacific Island	Employed	Rheumatology clinic
14	34	3 months	Pacific Island	Employed	Rheumatology clinic
15	34	8	Asian	Employed	Rheumatology clinic
16	54	24	NZ European	Employed	Rheumatology clinic
17	62	39	Asian	Retired	Rheumatology clinic
18	54	20	Asian	Retired	Rheumatology clinic
19	48	3	Asian	Unemployed	Rheumatology clinic
20	54	20	Asian	Employed	Rheumatology clinic

## Comfort’s number one

Women tended to have one to two pairs of retail shoes which they were able to wear, and they wore these constantly, with most reporting an inability to go barefoot for any length of time. Comfort was seen as the top priority, followed by fit, despite aesthetics being of high importance.

*“Comfort’s number one, and then colour, shape, the look of the shoe. I can’t anymore wear shoes with heels or anything like that at all, so I’m rather limited.”* - Participant 3, 71 years old.

Cost was commonly discussed by women and suitable retail shoes were generally associated with high cost, however, high cost was not a guarantee of a suitable shoe. Many women discussed financial pressures contributed to making bad choices in relation to footwear, and this led to anxiety about purchasing decisions.

Cushioning in the sole of the shoe was considered to be essential by all women, particularly under the heel and forefoot. Fixation and ease of application and removal were discussed by most, with many expressing difficulty with shoelaces and seeking footwear which could be applied easily without too much use of their hands.

*“Yeah, and a shoe that [I] can put on and take off easily, yeah that’s important as well. Because putting on shoes and taking off shoes is also quite a problem for me.”* - Participant 17, 62 years old.

Most women expressed a preference for a small heel, however many conceded that discomfort was a barrier to wearing such a style. A few women expressed a flexible sole and a non-slip outsole as preferable options. The weight of the shoe was discussed by many women, with a preference for lightweight shoes.

*“Yeah, I tried several shoes. I can’t wear heavy, I have to wear the light ones.”* - Participant 18, 54 years old.

In regards to fit, many women purchased their shoes a size larger to accommodate for the width of their forefoot or to accommodate toe deformities. This resulted in some women experiencing trips or falls as a result of their oversized shoes. Finding shoes with adequate width was also frequently reported as a barrier in purchasing retail footwear. Furthermore, the breathability and materials the shoes were made of were also closely examined by most women with a preference for soft leather and natural materials which were seen as more likely to breathe.

*“I always, [buy] leather shoes….. I would never wear plastic shoes, or cheap shoes, yeah…just because they’re a natural fibre…because you sweat in them, and your body sweats in synthetic or plastic shoes.”* - Participant 5, 64 years old.

Despite the stress and difficulties in obtaining comfortable and acceptable retail footwear that also maintained their identity as a woman, these women considered their footwear needs very carefully. There were common features that women identified as preferred, but overwhelmingly comfort was identified as a necessity.

## “I don’t want to wear Nana shoes, and so many comfy shoes are Nana shoes”

Many women expressed loss of identity and a sense of grief over the shoes which they were no longer able to wear since their RA diagnosis, and the loss of choice relating to purchasing retail footwear. This loss of choice in footwear contributed to a visual change in identity from who they were prior to their RA. The loss of choice was frequently expressed as constant compromise with regards to comfort versus aesthetics.

*“I can’t even wear heels anymore, can’t wear heels anymore. And [to find] a shoe that’s really comfortable is really hard.*”- Participant 10, 27 years old.

*“I don’t think I’ll ever go back to heels, I mean, I’ve worn them, and I used to have boots with heels, like before all this happened, but I just don’t’ think that’s a future for me.”* - Participant 11, 72 years old.

Furthermore, many women discussed footwear as a means of expressing themselves as a woman, and that they were no longer able to completely express themselves due to their limited choice in retail footwear. This was partially due to their limited shoe choice also dictating their clothing choices, which influenced their self-identity.

*“Frustrated that I kind of look at fashionable things and think that would be nice, or I’ve got a dress, it would be nice to have some pretty strappy sandals or something. And go well it’s just not going to work, because for a start I wouldn’t get my foot in them, but if my foot did go in them I just wouldn’t be able to walk in them, yeah. So, it’s frustrating, you feel like you compromise, everything’s compromised around being comfortable.”* - Participant 1, 44 years old.

Women of Asian background reported that it is culturally appropriate to remove shoes on entering another person’s home. These women would actively avoid social interactions knowing that removing shoes would be needed due to the difficultly in removing footwear and also the pain and embarrassment associated with being barefoot. Asian women with RA sought footwear which could easily put on and taken off, in particular, footwear with fastenings which allow for this.

*“And one thing is you can’t take the shoe off and on easily, yeah. So like we Asians, we usually want people to take off your shoe before you enter their house. Yeah, yeah so that’s a big problem, so that’s why I avoid going to friends, visiting friends, yeah….….even sometimes people invite me to their house for a party I would decline that. So that makes me a bit sort of, people say oh you’re anti-social or all kinds of remarks, or you’re not going to mix with us, or things like that, you know, some comment. So that’s a bit difficult.”* - Participant 17, 62 years old.

Women expressed a desire to wear footwear which were feminine, however, in most instances were unable to wear these types of shoes due to their RA-related foot pain and deformity. This was particularly evident when women would discuss dressing for special occasions, which they were unable to dress in a way that reflected their own femininity, which was dictated by their limitations in footwear choice.

*“It becomes a problem when you want to go to a wedding or something like that, or you want to go out for the evening, you’ve got a real problem about what footwear you might like to wear. Because I mean these are not exactly conducive to an evening out”.* - Participant 3, 71 years old.

Further, feelings of sadness were expressed about wanting to feel feminine.

*“Obviously I can’t wear heels, so it’s very hard to look elegant anymore.”* - Participant 7, 75 years old.

Feelings of embarrassment and self-consciousness were also frequently reported by women relating to their choice in retail footwear and body image. This was particularly evident in the younger women, and the women who were still in the workforce. These women felt that their footwear choices often needed to be justified to others, with some women requiring formal approval to wear joggers (trainers) in the workplace. Furthermore, negative emotions relating to premature ageing were expressed frequently by the younger women in relation to footwear choices.

*“I’m 44 so I don’t want to wear Nana shoes, and so many comfy shoes are Nana shoes. And yeah, you look in the fashion shops now and it’s just, well I can only look really.”* - Participant 1, 44 years old.

Restrictions in footwear choices had a huge impact emotionally in relation to how these women perceive themselves. This created sadness and other negative feelings.

## “Come live with my feet and you’ll understand”

Only half of the included participants had sought out podiatry care relating to their RA foot problems. Some women did not know what a podiatrist was, or did not understand how a podiatrist could help with their foot problems. When podiatry care was received, footwear advice was frequently not given at all, with many respondents unaware that podiatrists could provide footwear advice.

*“I wouldn’t have thought of a podiatrist to ask about shoes, to be honest………they seem to be more concerned with the actual feet rather than, or the nails and things rather than the shoe side of it.”* - Participant 8, 65 years old.

For those few women who had received footwear advice from podiatrists, the advice received was generally non-specific and vague. Furthermore, some women felt that their opinions were not heard by their podiatrist and that the advice they received was not tailored to them personally.

*“I think just in very general terms of wearing something that was comfortable on the feet and not putting too much pressure on points that might be sore. But nothing really specific, yeah just kind of wear something that’s comfortable and supportive. But that was about as far as we got.”* - Participant 1, 44 years old.

Further, for the women who were active in the workforce, they expressed that their peers did not understand their footwear choices and more broadly how this related to their RA disease status and foot pain and deformity. They expressed that their daily experience of living with foot pain was invisible, and something that could not possibly be understood by their peers, and this influenced their full participation in the workforce. Many felt judged by their footwear in the workplace which impacted on their self-esteem.

*“Gosh, your shoes look enormous, what are you wearing those huge shoes for today?”* - Participant 17, 62 years old.

*“Yeah [athletic shoes], literally the only shoes I have, so I wear them to work and get funny looks from people”.* - Participant 2, 56 years old

Most women expressed the retail shoe shopping experience was not enjoyable, and had negative experiences and lack of understanding by the retail staff when attempting to purchase retail footwear. Furthermore they expressed high levels of stress and anxiety associated with the experience. These feelings were sometimes further exacerbated by the retail staff who did not have any understanding of the RA disease process and the specific requirements for footwear. Feelings of embarrassment were also very frequent.

*“Well I’m a bit embarrassed about the [foot]…..I try and keep the sock on.”* - Participant 7, 75 years old

*“Watching like my nieces and my sisters buy shoes that I want to wear is, yeah, difficult to see. Like I used to wear those shoes, and now I can’t wear those shoes, it’s like playing that, playing in my head. And then like trying a shoe on, I get paranoid that people are looking at my feet.”*- Participant 10, 27 years old.

Only two participants had attempted online shoe shopping, with only one, a younger and recently diagnosed women, saying she would buy online again. Most of the women perceived the likelihood of having to return or exchange the shoes as a major barrier to buying online, and the inability to touch, feel and try the footwear on before committing to purchase. In the normal physical retail space, most participants had experiences of returning or exchanging footwear and most had described an experience of buying shoes at high cost which were unable to be worn, and still in their possession. Many women expressed feelings of anxiety and fear about purchasing footwear which may not be wearable.

*“I start to feel a bit like oh here we go again, they see me coming. I’m so nervous about paying a lot of money for shoes and then they don’t, you know, I have problems with them, because that’s what I’ve been doing, that’s what often happens” -* Participant 5, 64 years old.

*“I’ve actually bought shoes that I’ve tried on thinking that it was gonna work, but then buying it, and then regretting it when I get home. But it’s really difficult, yeah, hate it……Ah yip, price, price wise, like the more comfort shoes, it’s more expensive. Yeah, it’s so difficult, just hate, I hate shopping for shoes, that’s it.” -* Participant 10, 27 years old.

Women frequently expressed negative emotions relating to how others perceived them, their footwear, and furthermore how their peers behaved in response to their footwear choices. Of concern was that they felt that their opinions were not heard by their podiatrist and that the advice they received was not tailored to them personally.

## Discussion

The results of this study has provided deep insight into the key issues concerning the use of retail footwear in women with RA. The elicited themes demonstrate that the barriers for women with RA sourcing appropriate retail footwear are multi-faceted, and much more than the challenges related to finding footwear that has features that meet their own criteria of what is deemed contributes to comfort. However, the physical features of their retail footwear that achieve the desired comfort also create negative emotions. This has been found in previous studies [8–10]. However, these emotions are not just in relation to the appearance of the footwear but also a lack of understanding by others such as peers in the work place, retail staff in shoe shops but also in some cases by podiatrists.

The first theme, “Comfort’s number one”, related to the specific features of footwear that women with RA identified as desirable. Most of the features identified related to enhancing current function, and alleviating symptoms as a result of their RA disease process. Aesthetics were also deemed important, and preferable aesthetic features varied between the participants, but many women conceded that this was frequently compromised due to the needs of comfort. However not being able to choose a shoe which they found aesthetically pleasing was a large driver of dissatisfaction with their footwear. Cost of retail footwear in women with RA has not been previously discussed in the literature. The women in this study frequently discussed the high costs of suitable footwear which influenced purchasing decisions. Higher cost was generally associated with greater chance of comfort; however, this was not a guarantee, and women often felt nervous about making a significant financial commitment which may not result in a shoe which they would find comfortable.

In regard to specific shoe features, previous research has discussed that women with RA frequently prefer a sole which has cushioning [10], a finding which was echoed in this study. Cushioning was by far the feature that was most insisted upon by participants and highly influenced purchasing and wear decisions. Flexibility within the sole was also highlighted as preferable by a few of the women, which was unique to this study. This was an interesting preference and may relate to the reduced mobility in the foot commonly experienced by many women with RA deformity. Women may preference flexibility in a shoe in order to feel as though their joint mobility is not further impaired by the sole of a shoe*.* Whilst a flexible sole may be preferable for many women with RA, this may not be consistent with practitioners views, with more rigid soles with a forefoot rocker demonstrated to be more effective at reducing foot pain and improving function in people with RA [17]. However, the woman’s personal preferences should not be underestimated.

Material preferences have been discussed in previous literature, with leather being cited as desirable [10] which was also described by participants in the current study, however, breathability was additionally seen as a preferable feature in shoe materials by many. This may be due to the interviews being conducted over summer months, and the potential differences in summer climate in New Zealand, with previous research being conducted in the United Kingdom, summer in New Zealand necessitates footwear which is breathable. The weight of the shoe has previously been identified as challenging for women with RA [10], and women in this study similarly identified lightweight shoes as preferable. Women discussed that a heavy shoe adds additional strain to their legs and feet and was viewed as cumbersome. Fit was a key issue identified by most of the women in this study. Many of the participants identified finding retail shoes which accommodate their foot deformity as a result of the RA disease process, as challenging, with many women purchasing shoes in larger sizes in an attempt to accommodate their hallux-abducto valgus and digital deformities, which then results in a shoe which does not function appropriately. In cases where structural deformity is severe, therapeutic or bespoke footwear may be more beneficial, as retail footwear may not be able to accommodate such abnormal foot shapes. However, as previous research has highlighted [8], women with RA are hesitant to wear therapeutic or bespoke footwear, despite the benefits of fit and comfort, they would rather wear retail shoes with preferable aesthetic, which do not fit appropriately. In other chronic conditions, such as diabetes, footwear scales and tools exist which enable clinicians to objectively measure the experience of footwear [18]. However, in RA, there is no such tool.

The second theme- “I don’t want to wear Nana shoes, and so many comfy shoes are Nana shoes” - explored concepts of loss of identity, loss of choice, and feeling feminine. The feeling that there was a loss of choice in retail footwear since their RA diagnosis was expressed by most participants. Women with RA perceived footwear as a barrier to fully express their own identity and femininity. There was a sense of loss expressed when women reflected upon the choices in footwear which they felt the RA disease had essentially taken away from them. These findings align with previous work by Goodacre [9] and Naidoo [10]. However, unique to this study was the identification of cultural differences in the experience of footwear in women with RA in New Zealand. Due to our heterogeneous sample which was inclusive of a number of different ethnic backgrounds, we elicited an additional cultural barrier for Asian women with RA and their footwear experience. Previous research has identified that foot problems are highly prevalent in Asian populations with inflammatory arthritis [19]. The current study showed that Asian women with RA sought footwear which could easily be put on and taken off, in order to enter their home, or another person’s home. This has not previously been investigated and adds another layer of complexity for women with Asian backgrounds in sourcing suitable retail footwear.

The final theme, “Come live with my feet and you’ll understand”, explored a lack of understanding by peers, retailers and in some cases podiatrists in relation to their experience with footwear. Only half of participants had sought out podiatry care, and some women were unaware of the potential benefits of podiatry care [20]. An even smaller number had received useful advice on seeking appropriate retail footwear from podiatrists, however it was not clear from the results of this study that women were actively seeking footwear advice from their podiatrist. Previous research has shown that the retail shopping experience is not positive for women with RA [10], which was confirmed in this study. Of the women in this study who had sought out podiatry care, they reported not being given specific, targeted advice which could empower them to confidently purchase footwear with a greater chance of success. This potentially added to the negative experience of footwear purchase. A study by Hendry et al. [21] reported that people with RA perceive that many podiatrists lack the expertise in management of foot complications in RA with many expressing dissatisfaction that their primary complaints are frequently not addressed. Further education of podiatrists to not only manage foot complications in RA, but confidently recommend appropriate footwear, would be beneficial for patients with RA. Furthermore, educating women with RA to actively seek footwear advice from a podiatrist in order to gain knowledge on appropriate retail footwear features to aid in their decision making and improve their purchasing experience.

Furthermore, a previous study demonstrated that people with RA frequently wear retail shoes which are inappropriate and do not assist their disease state and furthermore could be contributing to foot pain [22] and one of the reasons women with RA wear unsuitable footwear is to avoid looking different to their peers [8, 9]. Participants in this study identified that their peers did not understand their unique needs in relation to footwear and this caused distress, particularly in the workplace. This led to some of the participants wearing shoes which were unsuitable and exacerbated their symptoms so that they did not stand out.

Finally, this study has identified that when women attempted to purchase retail shoes, most experience embarrassment about revealing their feet to shoe shop assistants. Many women discussed the fact that whilst the assistants were trying their best to help in most cases, they would frequently make negative comments on their foot deformities and make inaccurate assumptions based on inadequate knowledge of their underlying condition. This unique finding is of particular concern and it is clear that retail staff need increased understanding about the particular problems that women with RA experience in order to support appropriate choices. Further, increased knowledge of the desirable features of footwear as identified by these women is essential in maximise their potential to experience good foot health. There is scope for further training of footwear retail staff to gain knowledge in basic foot pathologies and to be able to accommodate people with special footwear needs, including women with RA. To this end, future research should focus on the development of a footwear suitability tool to support patients, retailers and podiatrists in footwear choices.

The results of this study elicited important information for the future development of a patient reported assessment measure to assess the experience of footwear in women with RA. In particular, the women’s perceptions of comfort, the psychosocial aspects of footwear, footwear appearance and specific features are key concepts, which will be integrated with expert opinion and the current literature base in the development of the assessment tool.

The findings of this study would be considered in light of some of the following limitations. We had a small sample of just 20 participants, however data saturation was achieved, and our intentionally heterogeneous population included a range of ages and different cultural backgrounds, which provided a diverse cross section of women with RA in New Zealand. Furthermore, recruitment included some patients with RA attending rheumatology podiatry clinics. Therefore, these findings may not reflect the experience of all women with RA.

## Conclusions

Women with RA have clear concepts about what features a shoe should have in relation to achieving comfort. Women with RA seek footwear which adequate cushioning, width, a flexible sole, lightweight, made from breathable materials and that are easy to apply. However, there is a constant compromise between achieving comfort and their feelings about their appearance and how they feel others perceive them. Of particular concern are the negative experiences with retail assistants in shoe shops and podiatrists. Footwear designers need to be mindful of creating styles that not only ensure comfort but also do not detract from femininity. Furthermore, both retail staff and podiatrists need increased awareness about the particular problems that women with RA experience in order to improve their overall experience with footwear and maximise foot health.

## Appendix

### Qualitative Interview Script

Warm Up Question: Tell me about your arthritis.

Initial Experience Question: Tell me about your experiences of shoes since being diagnosed with rheumatoid arthritis.

Dependent on the participant’s responses the following trigger questions will be asked to keep the dialogue flowing or in case they go off the topic of shoes:

- What is your experience of **buying** shoes from retail shops?
- How did you feel when you were buying the shoes?
- What is your experience of **wearing** retail footwear?
- How do you feel wearing shoes from retail shops?
- What do you look for when buying shoes from retail shops?
- What do you think you need from your footwear?
- How do the shoes available from retail shops meet or not meet your needs?
- What influences your decision about the shoes you choose?
- Have you sourced any information on the suitability of footwear from the web? (if so where? Was this helpful?)
- Have you ever received information or treatment related to shoes from a podiatrist?
- Are there any other aspects about shoes that you would like to tell me about?

If patients discuss a specific shoe in the past or present can ask: What effect have the shoes had on your ability to do things that you want to do?
